# Effect of Ultrasound and Chemical Cross-Linking on the Structural and Physicochemical Properties of Malanga (*Colocasia esculenta*) Starch

**DOI:** 10.3390/foods14152609

**Published:** 2025-07-25

**Authors:** Ana Sofía Martínez-Cigarroa, Guadalupe del Carmen Rodríguez-Jimenes, Alejandro Aparicio-Saguilán, Violeta Carpintero-Tepole, Miguel Ángel García-Alvarado, Ceferino Carrera, Gerardo Fernández Barbero, Mercedes Vázquez-Espinosa, Lucio Abel Vázquez-León

**Affiliations:** 1 Department of Analytical Chemistry, Faculty of Sciences, Institute for Viticulture and Agrifood Research (IVAGRO), University of Cadiz, Agrifood Campus of International Excellence (ceiA3), 11510 Puerto Real, Spain; d21020008@veracruz.tecnm.mx (A.S.M.-C.); ceferino.carrera@uca.es (C.C.); gerardo.fernandez@uca.es (G.F.B.); 2 Unidad de Investigación y Desarrollo en Alimentos (UNIDA), Campus Instituto Tecnológico de Veracruz, Tecnológico Nacional de México, Avenue Miguel Ángel de Quevedo 2779, Veracruz 91860, Mexico; guadalupe.rj@veracruz.tecnm.mx (G.d.C.R.-J.); miguel.ga@veracruz.tecnm.mx (M.Á.G.-A.); 3 Instituto de Biotecnología, Universidad del Papaloapan, Circuito Central 200, Col. Parque Industrial, Tuxtepec 68301, Mexico; aaparicio@unpa.edu.mx; 4 Tecnológico Nacional de México, Instituto Tecnológico de Tehuacán, Departamento de Ingeniería Química y Bioquímica, Libramiento Tecnológico SN, Tehuacán 75770, Mexico; violeta.ct@tehuacan.tecnm.mx; 5 SECIHTI-Tecnológico Nacional de México, Instituto Tecnológico de Tehuacán, Departamento de Ingeniería Química y Bioquímica, Libramiento Tecnológico SN, Tehuacán 75770, Mexico

**Keywords:** ultrasound, cross-linking, modified starch, malanga, non-conventional

## Abstract

Starch extracted from malanga (*Colocasia esculenta*) is a biopolymer with considerable industrial potential thanks to its high starch content (70–80% on a dry basis) and small granule size, which give it distinctive functional properties. To expand its applications in advanced processes such as encapsulation, it is necessary to modify its structural and physicochemical characteristics. This study evaluated the effects of ultrasound (US) and chemical cross-linking (CL) on the properties of this starch. US was applied at various times and amplitudes, while CL was performed using sodium trimetaphosphate and sodium tripolyphosphate, with sodium sulfate as a catalyst. US treatment reduced particle size and increased amylose content, resulting in lower viscosity and gelatinization temperature, without affecting granule morphology. Meanwhile, CL induced phosphate linkages between starch chains, promoting aggregation and reducing amylose content and enthalpy, but increasing the gelatinization temperature. The modified starches exhibited low syneresis, making them potentially suitable for products such as pastas, baby foods, and jams. Additionally, ultrasound modification enabled the production of fine starch microparticles, which could be applied in the microencapsulation of bioactive compounds in the food and pharmaceutical industries. These findings suggest that modified malanga starch can serve as a functional and sustainable alternative in industrial applications.

## 1. Introduction

Starch is an abundant, cost-effective, and non-toxic biopolymer that is produced by plants as an energy storage source. In recent years, there has been a growing interest in the extraction of starch from non-conventional sources, which are defined as raw materials that are not usually used for this purpose. The objective of these emerging studies is to revalorize natural resources by means of characterizing their physicochemical and functional properties. To date, many of these alternative starch sources remain insufficiently studied and underutilized at an industrial level, thereby limiting their integration into technological applications. Nevertheless, they offer a promising alternative to partially or totally replace conventional starches derived from agricultural crops such as potato (*Solanum tuberosum*), wheat (*Triticum aestivum*), and corn (*Zea mays*), which are primarily destined for human consumption [1,2,3]. The growing demand for alternative starch sources has encouraged research into the use of roots and tubers as potential substitutes for conventional crops [1,4]. Among them, malanga or taro (*Colocasia esculenta*), which is extensively cultivated in tropical regions due to its rapid growth and high yield, stands out. Mexico is one of the leading producers of taro worldwide, with an annual national production of 47,000 tons valued at MXN 252 million. Of this total, USD 21.7 million is exported to the United States. Veracruz stands out as the main producing state, contributing 43,500 tons per year [5,6]. The tuber contains 70–80% starch on a dry basis, with small granules (1–5 μm)—the smallest reported among tuber starches—which confer valuable functional properties. These characteristics make it suitable for use in a variety of industries, including the food, pharmaceutical, cosmetic, and textile sectors [7]. Nevertheless, native starch does not always exhibit the functional properties required for industrial applications, making modifications necessary to meet technological demands. As a result, a range of modification techniques have been examined. In recent years, modified starches have become widely used as functional ingredients or additives in a variety of food and beverage applications, as well as in industrial, cosmetic, and pharmaceutical formulations [8,9]. The modification of native starch can be achieved through a variety of processes, depending on the desired functional attributes and end-use. Chemical modification methods include acid hydrolysis, chemical cross-linking, esterification, and etherification [9,10,11], whereas physical modification methods involve techniques such as ultrasound, extrusion, high-pressure homogenization, high-energy milling, nanoprecipitation, and electrospraying [12,13,14,15,16].

Ultrasound (US) starch modification is an emerging physical technique that alters the structure and functional properties of starch without the use of chemical agents [17]. This method is based on the application of high-frequency ultrasonic waves, which induce cavitation phenomena within the medium in which the starch is dispersed. The formation and subsequent implosion of microbubbles generates localized areas of high temperature and pressure, which break polysaccharide chains and modify the granule size, morphology, and crystallinity [17,18]. Consequently, starches with enhanced properties can be obtained, such as higher solubility, lower swelling power, or reduced viscosity, which broaden their range of industrial applications. These changes are influenced by several factors, including ultrasound frequency and intensity, treatment time, temperature, and the botanical origin of the starch.

Chemical cross-linking (CL) is a widely used starch modification technique that enhances the functional stability of starch under demanding processing conditions such as elevated temperatures, agitation, or pH variations [11]. A variety of cross-linking agents are used for this purpose, including sodium trimetaphosphate (STMP), sodium tripolyphosphate (STPP), epichlorohydrin (ECH), and phosphoryl chloride (POCl_3_) [19]. These agents react with the hydroxyl groups of glucose chains within starch molecules to form covalent bonds between the amylose and amylopectin chains. STMP and STPP are the most commonly used among these in the food industry, as they are approved by the FDA. STMP forms phosphate ester linkages between hydroxyl groups, thereby generating a denser and more stable three-dimensional network. This modification produces starches that are more thermally resistant, more shear-stable, and lower in viscosity. This makes them especially useful in environments where prolonged structural integrity is a prerequisite. In the pharmaceutical industry, cross-linked starches are employed as excipients in controlled-release drug delivery systems and as fillers in tablet formulations. In the textile sector, they are used as thickeners in printing pastes, thereby providing enhanced resistance during the printing of fabrics [8]. In the paper industry, STMP-cross-linked starches have been shown to enhance the surface strength and finish quality of paper products [9]. These improvements mean that STMP can be used to modify starch for utilization in demanding industrial environments without compromising its biodegradability or renewable nature.

The modification of malanga starch by means of ultrasound (US) and chemical cross-linking (CL) represents a promising strategy for enhancing its functional properties and increasing its potential for industrial applications. The application of ultrasound treatment has been demonstrated to induce alterations in the granular structure, resulting in a reduction in particle size, thereby improving solubility. In contrast, chemical cross-linking has been shown to stabilize the polysaccharide matrix, thereby increasing its resistance to thermal processing, acidity, and shear forces. This makes it particularly suitable for application in the food and pharmaceutical industries. Looking ahead, these technologies will create new opportunities for the use of malanga starch as a functional biopolymer in advanced food applications, pharmaceutical matrices, and controlled-release systems, as well as in the development of biodegradable materials. This aligns with the growing trend towards sustainable, functional, and non-conventional ingredients. The objective of the present study was therefore to investigate the effect of US and CL on the functional properties of malanga starch.

## 2. Materials and Methods

### 2.1. Materials

Malanga (*Colocasia esculenta*) was collected from local cultivars located at 19°28′08″ N, 96°27′56″ W/19.468841666667, −96.465458055556 (Ignacio de la Llave, Veracruz, Mexico). Reagent-grade chemicals were purchased from Sigma Chemical Co. (St. Louis, MO, USA), and analytical-grade solvents from JT Baker (Mexico City, Mexico).

### 2.2. Starch Isolation

The isolation of starch was conducted following the method proposed by Aparicio-Saguilán et al. [20], with minor modifications. The malanga were peeled, cut into cubes measuring approximately 2 cm, and then ground using a disk mill (Agroxolo, Ecomaqmx, Heroica Puebla de Zaragoza, Mexico) (disk diameter: 24 cm; motor power: 2 hp). The resulting pulp was successively passed through stainless steel sieves (0.420 mm, 0.149 mm, 0.074 mm, and 0.044 mm). The material retained on the sieves was discarded, and the liquid fraction was left to settle at 20 °C for 12 h. The supernatant was then removed via decantation. The precipitate was washed three times via resuspension in water and then centrifuged (Heraeus Megafuge 16 R, ThermoFisher Scientific; Waltham, MA, USA) at 4000× *g* for 10 min at 20 °C. The sediment was then subjected to a drying process in a tray dryer (SUSESA; Veracruz, Mexico) at 45 °C until constant weight was achieved (approximately 12 h). The dried starch was then ground and sieved through a 0.44 mm mesh. The final product was stored at room temperature (25 °C) in airtight containers until further characterization and modification. This method was chosen because it helps to eliminate some of the oxalate and hydrocyanic acid in malanga, even though Vela-Gutiérrez et al. [21] reported that the levels of hydrocyanic acid in malanga pulp are not harmful to human health.

### 2.3. Ultrasound (US) Modification

Starch–water dispersions (20% *w*/*w*) were prepared and subjected to ultrasound treatment at various times (5, 15, 25, and 35 min) using a 750 W ultrasonic power and a frequency of 20 kHz (Sonics & Materials, Inc.; Newtown, CT, USA). Furthermore, the effects of amplitudes of 40%, 50%, and 60% were evaluated (Table 1). The temperature was maintained at 25 °C throughout the process using a water recirculation system. The modified starch was recovered by means of centrifugation at 4000× *g* for 10 min at 20 °C (Heraeus Megafuge 16 R, ThermoFisher Scientific; Waltham, MA, USA). The resulting sediment was dried in a tray dryer (SUSESA; Veracruz, Mexico) at 45 °C until constant weight was reached (approximately 14 h). The ultrasound-modified starches were stored in airtight containers until further characterization.

### 2.4. Chemical Cross-Linking (CL) Modification

Cross-linked starch was prepared following the method described by Woo & Seib [22], evaluating three levels of cross-linking agents and catalysts (Table 2).

### 2.5. Physicochemical Properties

#### 2.5.1. Viscosity Profile

A Discovery HR-2 hybrid rheometer (TA Instruments; New Castle, DE, USA) was used to determine the viscosity profile, equipped with a specific chamber and geometry for starch characterization (SPC SMART SWAF, Mod. 110533, New Castle, DE, USA), with a diameter of 32.40 mm and GAP of 500 µm (distance between the geometry and the base). A temperature ramp was applied to a solution of starch dispersed in water (10% *w*/*v*), following the method described by Aparicio-Saguilán et al. [20]. The temperature increased from 30 to 90 °C at 15 °C/min, held at 90 °C for 6 min, and then decreased from 90 to 30 °C at 30 °C/min. The results were recorded using Trios V4 software (TA Instruments; New Castle, DE, USA).

#### 2.5.2. Thermal Properties

The gelatinization temperature and enthalpy of the starch were determined via differential scanning calorimetry (DSC) using a TA Discovery DSC250 instrument (TA Instruments; New Castle, DE, USA), following the procedure described by Aparicio-Saguilán et al. [20]. The experiment was conducted using a heating program that ranged from 30 to 120 °C at a rate of 10 °C/min. This was performed under a constant nitrogen flow of 50 cm^3^/min. For each analysis, 2 mg of sample was weighed in hermetic aluminum pans (ref. 901684.901), to which 7 µL of deionized water was added. Subsequently, the pans were sealed and equilibrated for 30 min at room temperature prior to analysis. An empty aluminum pan was used as the reference sample. The results were recorded using Trios V4 software (TA Instruments; New Castle, DE, USA).

#### 2.5.3. Amylose Content

The amylose content was determined according to the method of Hoover & Ratnayake [23]. The starch was dissolved in 90% dimethyl sulfoxide (DMSO), stirred, and heated at 85 °C for 15 min. An aliquot was then mixed with an I_2_/KI solution and brought to a final volume of 50 mL. After standing for 15 min, absorbance was measured at 600 nm. A calibration curve was used (0.1–1.0 mg/mL amylose).

#### 2.5.4. Swelling Power and Solubility

The swelling power (SP) and water solubility (WS) were determined at different temperatures (60, 70, 80, and 90 °C) following the method described by Sathe & Salunkhe [24] and Aparicio-Saguilán et al. [20]. A 1% starch dispersion was prepared and heated for 30 min under constant stirring. The mixture was then cooled for 10 min and centrifuged at 4000× *g* for 10 min at 20 °C. The supernatant was separated, and the swollen granules were weighed. The supernatant was dried at 120 °C until constant weight, after which it was weighed. The SP and WS were calculated using Equations (1) and (2), respectively:

(1) $$\%\;\mathrm{Solubility}=\frac{\mathrm{Weight}\;\mathrm{of}\;\mathrm{soluble}\;\mathrm{starch}\;\left(g\right)}{\mathrm{Weight}\;\mathrm{of}\;\mathrm{sample}\;\left(g\;d.b.\right)}\;\times \;100$$

(2) $$Swelling\;Power=\frac{Weight\;of\;sediment\;\left(g\right)}{Weight\;of\;sample\;\left(g\right)-Weight\;of\;soluble\;starch}$$

#### 2.5.5. Refrigeration and Freezing Stability

The refrigeration and freezing stability of the samples were evaluated using the methods proposed by Aparicio-Saguilán et al. [20] and Eliasson & Ryang Kim [25]. Two dispersions were prepared using 0.25 g of starch and 5 mL of water each. These were subjected to a heating process at 85 °C for 15 min and then left to cool to room temperature. One sample was stored at 4 °C (refrigeration), and the other at −12 °C (freezing) for 24 h for each of them. Afterwards, both samples were centrifuged at 8000× *g* for 10 min. The liquid separated from the gel was measured. This procedure was repeated for 5 cycles, each consisting of 24 h. The percentage of syneresis was calculated using Equation (3):

(3) $$Syneresis\;\left(\%\right)\;=\frac{Weight\;of\;decanted\;liquid\;}{Weight\;of\;the\;sample\;before\;centrifugation\;}\times \;100$$

#### 2.5.6. Water Activity

The water activity of the samples was determined at 25 ± 1 °C using a water activity meter (Decagon Devices, Inc., AquaLab VSA; Pullman, WA, USA).

### 2.6. Morphological Analysis

The morphology of the starch granules was examined via scanning electron microscopy (SEM) using a JEOL JSEM 35CX microscope (Jeol Ltd., Tokyo, Japan) at an accelerating voltage of 20 kV [26]. The granule size distribution was assessed according to the method reported by Vázquez-León et al. [26] using a laser diffraction particle size analyzer (Malvern Instruments Ltd., Mastersizer 3000; Malvern, UK) equipped with a wet sample dispersion unit via immersion (Malvern Instruments Ltd., Hydro EV; Malvern, UK). The starches were dispersed in water until a light obscuration of 3 to 5% was achieved.

## 3. Results and Discussion

### 3.1. Starch Isolation Yield

The functional properties and applications of starches depend on both the botanical source and the extraction process. The purity of native malanga starch was found to be 97.19%, a value that is consistent with previously reported values for this tuber (97–99%) [8]. The yield of taro starch was found to be 19.83% on a wet basis. This yield was higher than those reported for Gros Michel banana (5.78% wb), Dominico Hartón (12.73% wb) [27], and jinucuil (7.63% wb) [20]. To date, the utilization of taro starch in commercial contexts remains non-existent; however, there is a discernible increase in the level of interest in the large-scale production of this substance, owing to its physicochemical and functional properties. These include its potential use as an ingredient in biodegradable packaging films, as a fat replacer, and for the encapsulation of ingredients and flavors, which is attributed to its small granule size. Additionally, this starch shows potential for use in pharmaceutical formulations, acting as an excipient and binder [8].

### 3.2. Physicochemical Properties of Modified Starches

#### 3.2.1. Viscosity Profile

When starch granules are heated in water, the double helices of amylopectin unwind, thereby exposing the hydroxyl groups within them. These groups subsequently interact with water through hydrogen bonding, resulting in an increase in viscosity. The viscosity profile of native taro starch is presented in Table 3, showing a maximum viscosity of 3.14 Pa·s, and the characteristic profile of starch [8,28,29]. The viscosity profiles of starches modified via US exhibited a significant difference (*p* < 0.05) in comparison to the native starch. Specifically, a decrease in maximum viscosity from 3.14 Pa·s to 2.40 Pa·s was observed. The reduction in the peak viscosity is attributed to structural changes induced via sonication. This phenomenon is attributable to the fragmentation of amylose and amylopectin chains caused by the action of ultrasonic waves [30]. The absence of significant changes in the maximum viscosity across the range of sonication times and amplitudes studied is attributed to molecular packing within the starch granule, specifically due to the double helices of amylopectin. Ultrasonication can exert greater mechanical force along the same spatial orientation of the chain, which is less effective in disrupting helical polysaccharides such as starches [17]. Another key factor is temperature control, which has been demonstrated to assist in the reduction in particle size [11,16]. This is achieved by preventing gelatinization and the subsequent collapse of the starch granule. Regarding the second viscosity peak, a reduction in retrogradation viscosity was observed in comparison to the native starch. This behavior has also been reported in relation to ultrasound modification in sweet potato starch [11] and rice [16]. The reduction in the retrogradation peak is associated with the fragmentation of amylose chains and the debranching of amylopectin, both of which are caused by the effects of ultrasonic waves [30]. Ultrasound can induce various chemical changes in polysaccharides, including depolymerization and reticulation, thereby diminishing their capacity to interact with water [17,31].

Starches modified via CL (Table 3) exhibit a different viscosity profile compared to native starch, suggesting that the cross-links contribute to stabilizing the granular structure of the starch by impeding its interaction with water [32,33]. It was observed that only two conditions retained noticeable viscosity peaks: the starch modified with a 30:70 STMP–STPP ratio and 0 g sodium sulfate, and the one modified with a 60:40 STMP–STPP ratio and 0 g sodium sulfate. This highlights the importance of adding sodium sulfate to facilitate effective cross-linking. The findings of this study suggest that chemical modification has the potential to significantly reduce the viscosity of native starch. This behavior is attributed to STMP acting as a cross-linking agent that reacts with the hydroxyl groups present in the amylose and amylopectin chains to form phosphate linkages. This agrees with the findings reported by Amaraweera et al. [34], who stated that cross-linking reactions induce the formation of intra- and intermolecular covalent bonds at random loci along the starch polymer chains. This reaction results in a denser and more structurally stable three-dimensional network. Consequently, the modified starch exhibits increased thermal resistance, improved shear tolerance, and reduced viscosity. These properties are particularly relevant in applications where high structural stability is required during processing or storage [35].

#### 3.2.2. Thermal Properties

**Figure 1:**
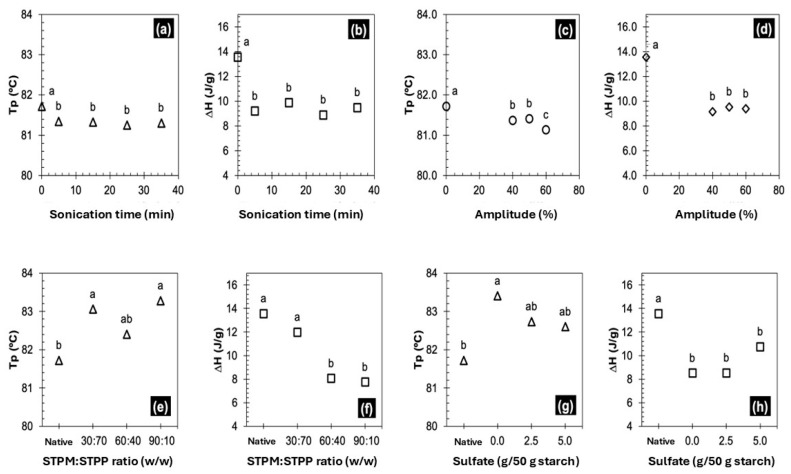
Main effects plot of gelatinization temperature (Tp) and enthalpy (∆H) of taro starch modified via US and CL. The studied US variables were sonication time (**a**,**b**) and amplitude (**c**,**d**). The CL conditions were STMP–STPP ratio (**e**,**f**) and catalyst amount (**g**,**h**). Values represent the mean of three replicates. Different letters within each graph indicate significant differences (*p* < 0.05).

The thermal properties of native and modified taro starches are presented in Figure 1. These results are consistent with the trends observed in the viscosity profiles (Table 3). A substantial decrease in gelatinization temperature was observed (Figure 1a,c). The aforementioned alterations are attributed to the disruption of the ordered double-helical structures and a decrease in crystalline regions, which consequently results in a lower dissociation temperature [11,31]. The effect of US on ΔH was not significant (*p* > 0.05) across the range of sonication times or amplitudes that were evaluated (Figure 1b,d). The reduction in the gelatinization temperature and the decrease in ΔH suggest that some chains were degraded and some double helices were broken during ultrasonic cavitation [11,31]. This behavior has also been reported in US-modified starches from cassava, sweet potato, and taro [11,13,30]. It may also be related to the irregularly shaped granules produced after US treatment. These results are consistent with those reported by Cui & Zhu [17]. In contrast, CL significantly affected (*p* < 0.05) both the gelatinization temperature and the enthalpy. A significant increase in the maximum temperature was observed (Figure 1e,g). The increase in gelatinization temperature that has been observed in CL-modified starch can be attributed to the degree of cross-linking or substitution—namely, the strengthening of intermolecular bonds between starch chains through the incorporation of phosphate groups [36]. Despite the decrease in enthalpy observed for the CL-modified starch (Figure 1f,h), this phenomenon can be attributed to the structural organization that is induced via cross-linking. This reduces the enthalpy due to the formation of bonds that reinforce the starch structure, thereby requiring elevated temperatures for gelatinization to occur [37]. CL modification also induces molecular damage to the starch structure, resulting in the formation of free water molecules, and, consequently, a decrease in crystallinity. However, it has been reported that the thermal properties—particularly the gelatinization and enthalpy—are associated with both the degree and organization of crystalline regions [38]. This finding is consistent with the results observed in this study through this chemical modification.

#### 3.2.3. Apparent Amylose Content

Table 4 shows the amylose content of both native and modified taro starches. The physical properties of starches are closely related to the amylose-to-amylopectin ratio [39]. The native starch exhibited a typical amylose–amylopectin ratio (25–75). However, in the case of US modification, an increase in amylose content was observed in comparison to the native starch. This phenomenon is attributed to the cavitation effect produced via the ultrasound treatment. This is due to the high shear forces that are generated during the process of sonication. These forces have been shown to promote the degradation of starch chains [17,33]. The amylose content of the modified starches is comparable to the values reported for hybrid corn starch [39] and can therefore be used as a reference point for normal corn starch [40]. In contrast, CL modification resulted in a decrease in amylose content. This is because the chemical treatment facilitates the formation of intra- and intermolecular covalent bonds between short starch chains (amylopectin branches and amylose chains) [11,34].

#### 3.2.4. Swelling Power and Solubility

**Figure 2:**
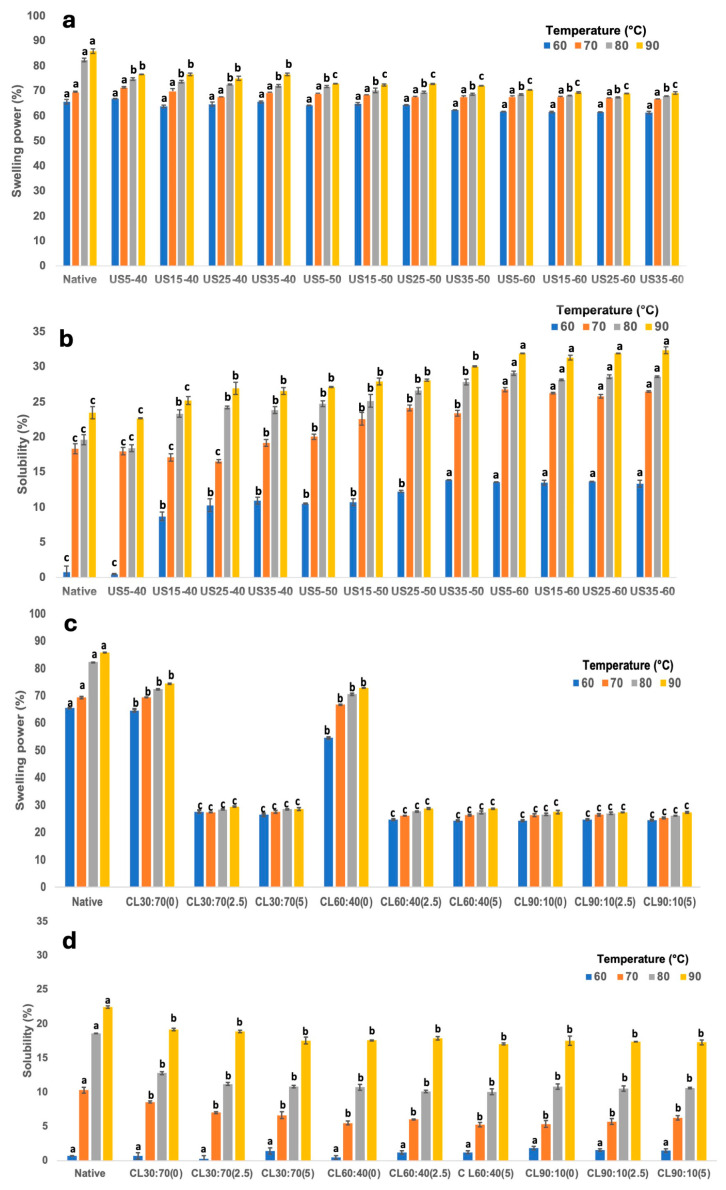
Swelling power and solubility of starch modified via ultrasound (**a**,**b**) and chemical cross-linking (**c**,**d**). Each bar color represents the temperature applied during the test. Different letters within bars of the same color indicate significant differences (*p* < 0.05). Values represent the means of three replicates.

Swelling and solubility are indicators of the gelatinization properties of starch granules. Swelling power is defined as the capacity of the granule to absorb water [41]. Figure 2 shows the behavior of the swelling power and solubility of the modified taro starches. It can be observed in Figure 2a that the starch granule begins to swell as the temperature increases across all samples, which is a typical behavior for starches. This swelling process is reflected in the corresponding viscosity values observed in Table 3. Starch modified via US exhibited lower swelling power than native starch due to sonication. Furthermore, it was observed that following a treatment period of 5 min at 50% amplitude (US5-50), no significant changes in the variables under investigation were detected. In contrast, the starch modified via CL (Figure 2c) did not exhibit the characteristic swelling behavior of native starches. Consequently, the viscosity profile exhibited by the sample was not consistent with that of native taro starch (Table 3). Regarding solubility, US-modified starch (Figure 2b) exhibited increased solubility in comparison to the native form. This increase can be attributed to the sonication-induced breakdown of amylopectin branches and amylose chains, resulting in a higher apparent amylose content after treatment (Table 4). Conversely, the CL-modified starch (Figure 2d) exhibited a lower percentage of solubility. This reduction can be attributed to the strong interactions with the phosphate groups [32,34]. Vamadevan & Bertoft [42] reported that the granular integrity of starch is a key factor in determining its swelling and solubility. As can be seen in the SEM images, no pores or cracks were present on the malanga starch granules. The presence of such pores or cracks would have facilitated water penetration into the granules, thereby promoting the hydration, swelling, and leaching of starch [43].

#### 3.2.5. Freeze–Thaw Stability

**Figure 3:**
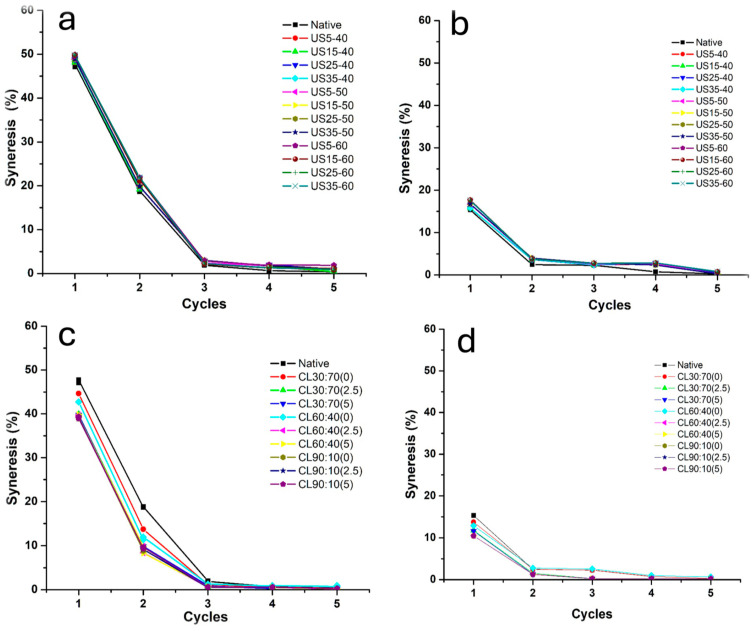
Syneresis across different cycles of refrigeration (**a**,**c**) and freeze–thaw treatment (**b**,**d**) of taro starch gels modified via US (**a**,**b**) and CL (**c**,**d**). Values represent the means of three replicates.

Figure 3 shows the freeze–thaw and refrigeration stability of gels made from taro starch modified using US (Figure 3a,b) or CL (Figure 3c,d) over five cycles. In the first cycle, under both refrigeration and freeze–thaw conditions, the highest syneresis values were observed for both types of modifications. However, starting from the third refrigeration cycle and the second freeze–thaw cycle, no significant increase in syneresis percentage was observed, which is in contrast with the findings reported in other studies [44,45]. This behavior suggests that the gels obtained from US- and CL-modified starch exhibit greater stability under refrigeration and freeze–thaw processes. A high amylopectin content can produce gels with a low tendency toward retrogradation, as evidenced by the storage stability results for starches modified via US and CL [44].

#### 3.2.6. Water Activity (*a*_w_)

The *a*_w_ value obtained for malanga starch was 0.43 (Table 5), which is consistent with the value reported for this tuber in the literature [8]. Conversely, the starches modified via US exhibited no significant differences among themselves under the conditions studied. Their values ranged from 0.27 to 0.35, which is lower than that of the native starch. This phenomenon may be attributed to a minimal reorganization of the chains, resulting in the formation of a network that captures previously unbound water molecules. In the case of starches modified via esterification (EQ), the *a*_w_ values ranged from 0.30 to 0.49 across the different STMP and STPP levels evaluated. The lowest *a*_w_ value is attributed to the formation of intramolecular interactions between the phosphate groups and the starch matrix, which is induced via EQ and leads to a reduction in free water [32]. In contrast, the highest *a*_w_ value is associated with molecular damage to the starch structure caused by EQ, resulting in the release of unbound water molecules. This phenomenon is supported by the enthalpy decrease observed in Figure 1f,h. Regardless of the type of modification (US or CL), the water activity (*a*_w_) values remained below 0.45, thereby contributing to particle stability by making the starch less susceptible to agglomeration or hardening during storage [46,47].

### 3.3. Morphological Analysis

#### 3.3.1. Particle Size Distribution (PSD)

**Figure 4:**
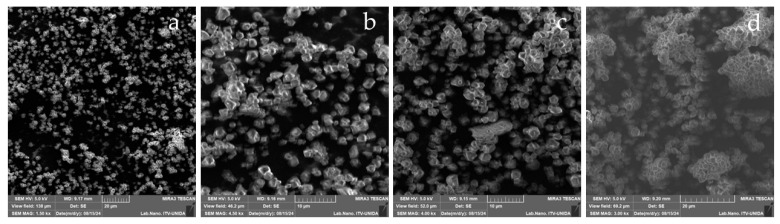
Scanning electron microscopy (SEM) images of native taro starch (**a**,**b**), ultrasound-modified starch, (**c**) and chemically cross-linked starch (**d**). SEM MAG: (**a**) 1.50 kx; (**b**) 4.50 kx; (**c**) 4.00 kx; and (**d**) 3.00 kx.

Table 6 shows the DV (50) values for native starch and starches modified via US and CL. It can be observed that the native starch exhibits a characteristic DV (50) value ranging between 1 and 20 µm [8]. In contrast, the US-modified starches demonstrate a reduction in particle size, with the smallest size (5 µm) observed at 60% ultrasound amplitude. Moreover, this finding suggests that the modification time does not appear to significantly influence the particle size. This size reduction has been widely reported as a primary effect of ultrasound treatment modification [11,13,16]. The US35-60 modification exhibited an increase in the DV50 value in comparison to the native starch. This increase is attributed to the fragmentation of the granules into smaller particles because of the cavitation induced via ultrasound (US) treatment. Nevertheless, these particles exhibited a tendency to agglomerate, forming larger structures and resulting in an increase in the mean particle size (DV50). This behavior has previously been reported for other starch sources modified via ultrasound, such as sweet potato [11] and cassava [30]. In the case of CL modification, an increase in particle size distribution was observed. This phenomenon is attributed to the inter- and intramolecular interactions between the starch molecules and the phosphate groups [30]. As can be seen in the SEM image (Figure 4d), there is clear evidence of particle agglomeration, which is a consequence of chemical modification. 

#### 3.3.2. Optimal Conditions

According to the results obtained, the optimal conditions for the ultrasound (US) treatment were US5-60 (5 min, 60% amplitude). This treatment promoted an increase in amylose content, which in turn improved the starch’s solubility. Additionally, a decrease in gelatinization temperature and enthalpy was observed, along with significant reductions in particle size, swelling power, and viscosity. No significant differences were detected when increasing the ultrasound exposure time, suggesting that this condition is efficient. This approach represents a more sustainable alternative, as it is environmentally friendly and allows for reduced processing times and energy consumption. In the case of CL, the optimal conditions were 60:40 STMP–STPP (*w*/*w*) and 2.5 g of catalyst. Under these conditions, a decrease in amylose content was observed, which also led to a reduction in starch solubility. Significant decreases in swelling power and viscosity were also recorded. Furthermore, the modified starch exhibited greater thermal stability, as evidenced by an increase in the gelatinization temperature. No significant differences were observed when increasing the concentration of cross-linking agents and catalyst, suggesting that the treatment is effective with lower amounts of reagents, thus contributing to raw material savings and reduced production costs.

#### 3.3.3. Scanning Electron Microscopy (SEM)

After the partial characterization of the starches modified via US and CL, a condition was selected for each modification based on the criteria described in the previous section (Section 3.3.2).

Figure 4 shows the morphology of native taro starch and starch modified via US and CL. It has been observed that the native taro starch exhibits a characteristic polygonal morphology [8], consisting of small granules (Figure 4a,b), which is consistent with the particle size distribution observed in Table 6. In the case of US-modified starch (Figure 4c), the granules appear intact, indicating that the ultrasound treatment did not cause granule rupture. This finding is consistent with the observations reported by Rahaman et al. [48] for corn and cassava starch, where SEM analysis revealed that the ultrasound-treated samples exhibited granules with smooth, crack-free surfaces. This preservation of granule integrity is attributed to the absence of gelatinization during the process, which would have led to granule collapse. This level of control was achieved by maintaining the temperature during sonication [11]. The results of the SEM and PSD analyses confirmed that US broke the starch granules down into smaller particles. This effect of ultrasound has previously been reported in the literature for oat, taro, and rice starches [13,16,47]. This phenomenon is due to the cavitation induced via ultrasonic waves. Conversely, CL-modified starch exhibits evidence of agglomeration, resulting in an increased particle size (Figure 4d). This is attributed to the interactions between the phosphate groups and the hydroxyl groups of the amylose and amylopectin chains in taro starch [32,34]. The visual evidence also confirmed the effect of chemical modification, whereby intra- and intermolecular interactions with phosphate groups increased particle size through cross-linking [32,35].

### 3.4. Chemometric Analysis

**Figure 5:**
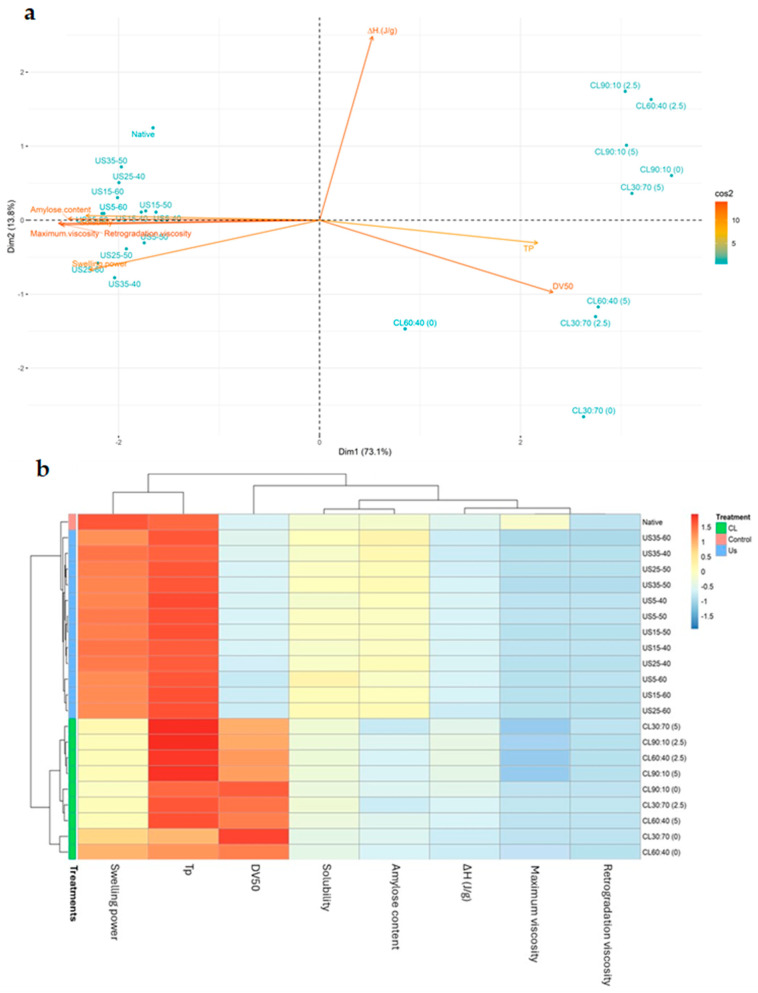
Chemometric analysis of the effects of modifications on the functional properties of malanga starch. (**a**) Principal component analysis (PCA) and (**b**) Heatmap.

Figure 5 shows the chemometric analysis of the modifications via US and CL. It illustrates how each modification influences the functional properties of taro starch, as well as the manner in which these functional properties are related to one another. In the Principal Component Analysis (PCA) (Figure 5a), PC1 explains 73.1% of the data variability, and PC2 explains a further 13.8%. Together, these two dimensions account for 86.9% of the total variability in the data. It is evident that both modifications affect the functional properties in different ways, thus enabling a clear separation between them based on the properties that have the greatest influence, namely, maximum viscosity, swelling power, amylose content, and retrogradation viscosity. Conversely, enthalpy does not allow for differentiation between the effects of US and CL. However, enthalpy, in conjunction with gelatinization temperature and DV50, reveals the effect of the chemical modification (CL) according to the mixtures under study.

Similarly, in the Heat Map (Figure 5b), the separation in the effect of both modifications on the functional properties is also evident, as they are found in two different clusters. Likewise, it is also observed that both modifications influence the native starch, which is found in a separate cluster. Furthermore, the various clusters shown in the upper part of the graph indicate how the properties relate to one another based on their similarities and differences. Finally, each color indicates the effect of the modifications on the properties of taro starch. Likewise, the darker the color, the greater the intensity of the effect.

Focusing on each property, it can be observed, based on color, that the native starch has the highest swelling power, followed by the US modifications and finally the CL modifications, as previously reported in Figure 2. In the case of gelatinization temperature, the highest values are obtained through chemical modification, while the lowest corresponds to the native starch, as shown in Figure 1. Regarding the particle size distribution (DTP), CL forms aggregates with a higher DV50, while smaller particle sizes are observed in the US and native starch modifications, as reported in Table 6. In terms of solubility, higher values are observed with the US modification, with no significant differences compared to native starch at 90 °C, as illustrated in Figure 2. The CL modification exhibited a reduced amylose content, while it increased in the US case, as detailed in Table 4. As for enthalpy, it was found that its value decreased in both modifications when compared to the native starch. The decrease was more pronounced in the CL modification, as reported in Figure 1. Finally, regarding viscosity and retrogradation peak, a decrease in their values is also observed, being more significant in the case of chemical modification, as demonstrated in Table 3.

As was stated in the preceding discussion, the PCA and Heat Map provide confirmation of the effect of each modification on the physicochemical properties of the starch. If the aim is to preserve the functional characteristics of starch, US is a more conservative approach. However, an increase in both amplitude and time was found to have a significant effect compared to native starch. This is due to the structural changes induced via sonication [30]. In contrast, if the objective is to structurally modify the starch (for example, for applications requiring greater thermal resistance or lower viscosity), CL is more effective, since the chemical modification enables the formation of intra- and intermolecular covalent bonds with the starch chains, thereby providing enhanced stability [11].

## 4. Conclusions

This study demonstrates that taro starch (*Colocasia esculenta*), a non-conventional botanical source, possesses significant potential for industrial applications, particularly within the food sector, including products such as pastes, baby foods, and jams. Starch modification—achieved through either physical methods like ultrasonication or chemical treatments such as cross-linking—enables the adjustment of functional properties to meet specific technological requirements, offering notable advantages over native starch.

Both modifications may also be suitable for high-temperature processing applications, if starch gelatinization is not required, due to the altered gelatinization temperatures observed. Ultrasound modification enabled the production of fragments of broken starch granules within the micrometer range, which may be suitable for the microencapsulation of bioactive compounds in food and pharmaceutical formulations. Regarding chemical cross-linking, the modified starch did not exhibit the typical viscosity profile of native starch, likely due to the formation of intra- and intermolecular interactions with phosphate groups.

Moreover, the use of alternative sources such as taro contributes to the diversification of raw materials, reducing reliance on conventional crops like corn and potato.

## Figures and Tables

**Table 1:** Experimental design used for the US treatment of malanga starch.

Code	Amplitude (%)	Time (min)
Native	-	-
US5-40	40	5
US15-40	40	15
US25-40	40	25
US35-40	40	35
US5-50	50	5
US15-50	50	15
US25-50	50	25
US35-50	50	35
US5-60	60	5
US15-60	60	15
US25-60	60	25
US35-60	60	35

Temperature was maintained constant at 25 °C, starch–water dispersions (20% *w*/*w*).

**Table 2:** Experimental design used for the chemical cross-linking of native malanga starch.

Independent Variables	Evaluated Levels		
STMP–STPP ratio (*w*/*w*) *	30:70	60:40	90:10
STMP (g per 50 g of starch)	0.9	1.8	2.7
STPP (g per 50 g of starch)	2.1	1.2	0.3
Sodium sulfate (g per 50 g of starch)	0	2.5	5

* The actual mass quantities used to achieve these proportions are specified in the following rows (STMP and STPP). The STMP–STPP mixture (*w*/*w*) was added at a total amount of 3 g per 50 g of starch.

**Table 3:** Viscosity profiles of aqueous dispersions of taro starch modified via US and CL (solid concentration of 10% *w*/*w*).

US-Treated Sample	Maximum Viscosity (Pa·s)	Retrogradation Viscosity (Pa·s)	CL-Treated Sample	Maximum Viscosity (Pa·s)	Retrogradation Viscosity (Pa·s)
Native	3.14 ± 0.10 ^a^	2.96 ± 0.01 ^a^	Native	3.14 ± 0.10 ^a^	2.96 ± 0.01 ^a^
US5-40	2.69 ± 0.08 ^b^	2.93 ± 0.00 ^b^	CL30:70 (0)	0.77 ± 0.00 ^c^	0.84 ± 0.02 ^c^
US15-40	2.65 ± 0.04 ^b^	2.77 ± 0.00 ^e^	CL30:70 (2.5)	0.14 ± 0.00 ^d^	0.25 ± 0.00 ^d^
US25-40	2.56 ± 0.09 ^b^	2.85 ± 0.00 ^c^	CL30:70 (5)	0.04 ± 0.00 ^g^	0.04 ± 0.02 ^g^
US35-40	2.47 ± 0.09 ^b^	2.50 ± 0.00 ^k^	CL60:40 (0)	1.77 ± 0.04 ^b^	1.54 ± 0.01 ^b^
US5-50	2.56 ± 0.09 ^b^	2.79 ± 0.00 ^d^	CL60:40 (2.5)	0.04 ± 0.00 ^g^	0.05 ± 0.01 ^g^
US15-50	2.51 ± 0.08 ^b^	2.73 ± 0.00 ^f^	CL60:40 (5)	0.03 ± 0.00 ^h^	0.05 ± 0.02 ^g^
US25-50	2.48 ± 0.09 ^b^	2.65 ± 0.00 ^g^	CL90:10 (0)	0.08 ± 0.00 ^e^	0.14 ± 0.00 ^e^
US35-50	2.48 ± 0.09 ^b^	2.63 ± 0.00 ^i^	CL90:10 (2.5)	0.06 ± 0.00 ^f^	0.14 ± 0.00 ^e^
US5-60	2.40 ± 0.00 ^b^	2.64 ± 0.00 ^h^	CL90:10 (5)	0.04 ± 0.00 ^g^	0.10 ± 0.00 ^f^
US15-60	2.42 ± 0.08 ^b^	2.77 ± 0.00 ^e^			
US25-60	2.50 ± 0.10 ^b^	2.73 ± 0.00 ^f^			
US35-60	2.48 ± 0.09 ^b^	2.58 ± 0.00 ^j^			

Values represent the means of two replicates ± standard deviation. Different letters within a column indicate significant differences (*p* < 0.05).

**Table 4:** Amylose content of taro starches modified via ultrasound (US) and chemical cross-linking (CL).

US-Treated Sample	Amylose Content (%)	CL-Treated Sample	Amylose Content (%)
Native starch	24.81 ± 0.23 ^f^	Native	24.81 ± 0.23 ^a^
US5-40	27.58 ± 2.15 ^ef^	CL30:70 (0)	14.95 ± 0.73 ^b^
US15-40	28.10 ± 0.53 ^e^	CL30:70 (2.5)	2.32 ± 0.00 ^d^
US25-40	31.21 ± 0.53 ^cd^	CL30:70 (5)	2.66 ± 0.48 ^d^
US35-40	34.15 ± 0.23 ^abc^	CL60:40 (0)	10.97 ± 0.00 ^b^
US5-50	26.71 ± 0.05 ^ef^	CL60:40 (2.5)	7.51 ± 2.93 ^c^
US15-50	27.40 ± 0.53 ^ef^	CL60:40 (5)	10.10 ± 0.24 ^c^
US25-50	31.56 ± 0.05 ^bcd^	CL90:10 (0)	9.41 ± 0.24 ^c^
US35-50	34.33 ± 0.05 ^ab^	CL90:10 (2.5)	9.07 ± 0.24 ^c^
US5-60	26.37 ± 0.05 ^ef^	CL90:10 (5)	7.34 ± 1.22 ^c^
US15-60	29.13 ± 0.05 ^d^		
US25-60	31.73 ± 2.15 ^bcd^		
US35-60	37.26 ± 0.23 ^a^		

Values are the means of two replicates ± standard deviation. Different letters within a column indicate significant differences (*p* < 0.05).

**Table 5:** Water activity of starches modified via ultrasound (US) and chemical cross-linking (CL).

US-Treated Sample	*a*_w_	CL-Treated Sample	*a*_w_
Native	0.430 ± 0.010 ^a^	Native	0.430 ± 0.010 ^a^
US5-40	0.272 ± 0.003 ^b^	CL30:70 (0)	0.493 ± 0.000 ^a^
US15-40	0.285 ± 0.004 ^b^	CL30:70 (2.5)	0.385 ± 0.001 ^c^
US25-40	0.280 ± 0.002 ^b^	CL30:70 (5)	0.312 ± 0.001 ^d^
US35-40	0.297 ± 0.001 ^b^	CL60:40 (0)	0.422 ± 0.001 ^bc^
US5-50	0.295 ± 0.006 ^b^	CL60:40 (2.5)	0.300 ± 0.001 ^d^
US15-50	0.312 ± 0.069 ^b^	CL60:40 (5)	0.446 ± 0.003 ^ab^
US25-50	0.312 ± 0.004 ^b^	CL90:10 (0)	0.428 ± 0.000 ^bc^
US35-50	0.314 ± 0.003 ^b^	CL90:10 (2.5)	0.304 ± 0.000 ^d^
US5-60	0.297 ± 0.000 ^b^	CL90:10 (5)	0.393 ± 0.000 ^c^
US15-60	0.270 ± 0.002 ^b^		
US25-60	0.301 ± 0.000 ^bc^		
US35-60	0.301 ± 0.000 ^bc^		

Values are expressed as the means of two replicates ± standard deviation. Different letters within a column indicate statistically significant differences (*p* < 0.05).

**Table 6:** Particle size distribution of starches modified via ultrasound (US) and chemical cross-linking (CL).

US-Treated Sample	DV50 (µm)	Span	CL-Treated Sample	DV50 (µm)	Span
Native	11.10 ± 0.10 ^bcd^	11.25 ± 0.06 ^d^	Native	11.10 ± 1.66 ^f^	124.66 ± 1.53 ^a^
US5-40	9.83. ± 0.55 ^de^	11.13 ± 0.57 ^d^	CL30:70 (0)	124.33 ± 2.88 ^a^	124.33 ± 0.57 ^b^
US15-40	8.66 ± 1.04 ^efg^	10.99 ± 0.86 ^d^	CL30:70 (2.5)	75.83 ± 3.06 ^c^	165.66 ± 1.53 ^c^
US25-40	8.80 ± 0.47 ^efg^	11.07 ± 0.54 ^d^	CL30:70 (5)	56.1 ± 2.19 ^e^	152.33 ± 0.58 ^c^
US35-40	11.95 ± 1.00 ^bc^	10.72 ± 0.82 ^d^	CL60:40 (0)	88.66 ± 1.33 ^b^	190.00 ± 1.00 ^c^
US5-50	9.17 ± 0.49 ^def^	14.41 ± 0.86 ^bc^	CL60:40 (2.5)	63.53 ± 0.20 ^d^	154.33 ± 1.53 ^c^
US15-50	9.99 ± 0.59 ^cde^	16.54 ± 0.95 ^ab^	CL60:40 (5)	72.33 ± 2.30 ^c^	154.33 ± 1.58 ^c^
US25-50	10.03 ± 0.40 ^cde^	12. 25 ± 0.49 ^cd^	CL90:10 (0)	86.3 ± 0.88 ^b^	184.00 ± 1.73 ^c^
US35-50	12.73 ± 1.27 ^b^	12.19 ± 1.45 ^d^	CL90:10 (2.5)	56.83 ± 2.20 ^e^	149.33 ± 0.58 ^c^
US5-60	5.40 ± 0.22 ^h^	17.27 ± 0.53 ^a^	CL90:10 (5)	60.3 ± 0.26 ^de^	156.00 ± 1.00 ^c^
US15-60	7.44 ± 0.90 ^fgh^	14.76 ± 0.59 ^b^			
US25-60	6.99 ± 0.54 ^gh^	16.01 ± 0.67 ^ab^			
US35-60	15.64 ± 0.42 ^a^	11.55 ± 0.26 ^d^			

Values are expressed as the means of two replicates ± standard deviation. Different letters within a column indicate statistically significant differences (*p* < 0.05).

## Data Availability

The original contributions presented in this study are included in the article. Further inquiries can be directed to the corresponding authors.

## Abbreviations

The following abbreviations are used in this manuscript:

US	Ultrasound
CL	Cross-linking
STMP	Sodium Trimetaphosphate
STPP	Sodium Tripolyphosphate
ECH	Epichlorohydrin
POCl_3_	Phosphoryl Chloride
FDA	Food and Drug Administration
DSC	Differential Scanning Calorimetry
SP	Swelling Power
WS	Water Solubility
SEM	Scanning Electron Microscopy
PSD	Particle Size Distribution
Tp	Gelatinization Temperature
∆H	Enthalpy
PCA	Principal Component Analysis
